# Mechanical, Thermal and Morphological Study of Bio-Based PLA Composites Reinforced with Lignin-Rich Agri-Food Wastes for Their Valorization in Industry

**DOI:** 10.3390/polym16172462

**Published:** 2024-08-29

**Authors:** Belén Soriano-Cuadrado, Mᵃ Ángeles Fontecha-Cámara, María Mañas-Villar, Irene Delgado-Blanca, María Dolores Ramírez-Rodríguez

**Affiliations:** Andaltec, Plastic Technological Center, 23600 Martos, Spain; mangeles.fontecha@andaltec.org (M.Á.F.-C.); maria.manas@andaltec.org (M.M.-V.); irene.delgado@andaltec.org (I.D.-B.); maria-dolores.ramirez@andaltec.org (M.D.R.-R.)

**Keywords:** lignin, PLA, composite, waste recovery

## Abstract

This study investigates the performance of different poly(lactic acid) (PLA) composites incorporating agri-food waste additives and commercial lignin, comparing their properties with those of virgin PLA. The following composites were prepared using a single-screw extruder: PLA with 20% rice husk, PLA with 20% wheat straw and PLA with 20% olive pit. Additionally, PLA was blended with commercial lignin at the maximum feasible proportion using the same methodology. The resulting composites were injection-molded into specimens for analysis of their mechanical, thermal and morphological behavior. The primary objectives were to assess the dispersion of the additives within the PLA matrix and to evaluate the mechanical properties of the composites. The results indicate that the addition of high percentages of agricultural residues does not significantly compromise the mechanical properties of the composites. Notably, in the case of the PLA with 20% rice husk composite, the elastic modulus surpassed that of virgin PLA, despite the evident heterogeneity in filler particle sizes. It was feasible to incorporate a higher percentage of agricultural residues compared to commercial lignin, attributed to the larger volume occupied by the latter.

## 1. Introduction

In recent years, there has been a growing concern about the scarcity of materials and, above all, about environmental conservation. This is due to the current shortage of materials as a result of the depletion of oil reserves and/or greenhouse gas emissions caused by the misuse of these materials [1]. This use has generated a high “burden” on the climate, which has led companies and businesses, as well as consumers, to actively seek more environmentally friendly products for marketing and future purchase [2].

As a result, over the years, novel bio-based polymers have been discovered in an attempt to address the gradual depletion of petroleum-based resources. Additionally, the high carbon footprint of fossil materials and the consequent increase in pollution, coupled with growing global environmental awareness, have shifted focus towards these bio-based polymers and their development [1,3].

Bioplastics are polymers produced from a renewable or natural source [1]. Among the most environmentally friendly options, poly(lactic acid) (PLA) stands out as a prominent bio-based polymer. PLA is a bio-based thermoplastic aliphatic polyester characterized by properties such as biocompatibility, compostability and good processability, making it a viable substitute for conventional polymers [4].

In addition to its properties of high strength and elastic modulus, this bio-based polymer is considered biodegradable because, upon hydrolysis and subsequent bacterial attack, the high-molecular-weight polyester chains are fragmented and these fragments (molecular weight of approximately 10,000 Da) are digested and completely decompose into CO_2_ and water [1,5,6].

It is synthesized by the polymerization of lactic acid, generally obtained by fermentation of starchy agri-food materials (e.g., maize, wheat, sugar cane [7]). However, inherent weaknesses in its properties, such as brittleness and low elongation at break due to the stiffness of its main chain, limit its applicability in certain contexts [8].

To overcome the limitations of PLA, many authors have used reinforcements, a very common practice that we can already find commercially for materials such as polypropylene in automotive applications. The use of biofibers has become increasingly important. This is due to the versatility of their eco-friendly design and the low cost of fibers that, unlike synthetic fibers, are organic and sustainable, thus creating cutting-edge materials [2]. For all these reasons, natural fiber-reinforced biocomposites have attracted increasing attention from researchers as these natural fibers would enable a low cost and provide good mechanical properties and lightness to the composite material [9]. 

Another point to consider is the overpopulation levels reached in recent decades, which has generated a high demand for food. As a direct consequence, production systems have led to an excessive use of natural resources. As a result, waste management has become an arduous task due to the high volume of waste. However, the circular economy is playing an increasingly important role by trying to replace end-of-life with material recovery, thus achieving increasingly sustainable development that provides value-added products. Although this problem exists worldwide, Spain is the leading European producer of vegetables, fruit and olives, although it also plays a significant role as a producer of pulses, cereals, sugar cane and tubers [10]. 

A high percentage of food waste consists of natural fibers, themselves composed of cellulose, hemicelluloses and lignin [11]. Authors such as Roostazadeh and Behzad have provided information on the use of starches, which have resulted in undesirable mechanical and thermal properties. However, they offer as an alternative the incorporation of lignin and lignin-rich residues to mitigate this problem [12]. 

**Figure 1:**
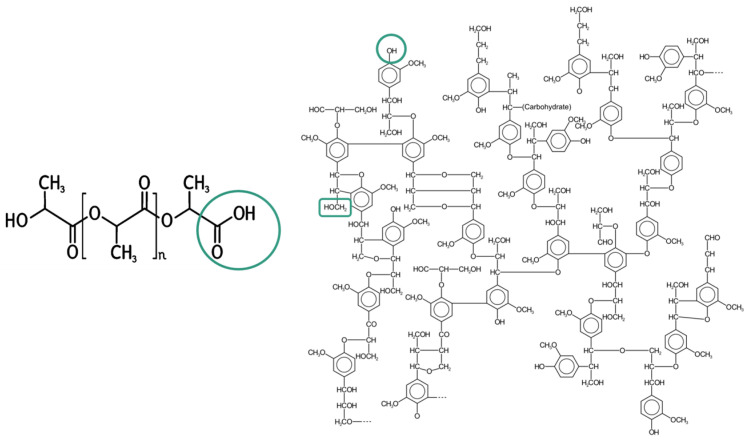
Hydroxyl and carboxylic functional groups in the structure of lignin and PLA that can form hydrogen bonds.

Lignin is the most abundant natural polymer after cellulose and has a high potential to reduce resource constraints. It can impart antioxidant and antibacterial properties to composites, making it attractive for applications in food packaging, agriculture and even biomedicine. Despite its complex branched structure and composition, lignin is an ideal candidate to interact with PLA due to its hydroxyl groups, which can form hydrogen bonds with the carboxyl groups of PLA [13], as shown in Figure 1.

In this study, we considered incorporating lignin-rich agri-food wastes such as olive pits, wheat straw and rice husks, extracted in a previous study [14], to reinforce the PLA polymer matrix. The rationale for this decision is elaborated in the following paragraphs.

Olive pits are a byproduct of olive oil extraction and, due to their physicochemical properties and calorific value, represent the most commercially valuable waste from the industrial olive sector [15]. Their heat of combustion makes them excellent candidates for biofuel use. However, their chemical composition—37.5% cellulose, 26% hemicellulose and 21.5% lignin—has attracted interest for their potential as reinforcements in composite materials [16].

Wheat straw is another abundant and low-cost waste. Historically underutilized, it is now recognized as a potential reinforcement for PLA-type materials, as noted by authors like Nyambo et al. [17]. This utilization not only helps maintain the CO_2_ balance but also reduces emissions associated with polyolefins and incentivizes farmers. Zhang et al. [18] have studied its lignin content, finding that dry wheat straw contains 11–26% lignin, with hydroxyl groups capable of forming hydrogen bonds with PLA, enhancing compatibility. 

Similarly, rice husks, an industrial byproduct of the milling process, present a significant disposal challenge, especially in top rice-producing countries like China, India and Indonesia, and globally reach up to 150 million tonnes annually. Previously used as fuel due to their low nutrient content and high lignin content (25–30%), rice husks are now being explored for their potential as reinforcements in biologically derived polymeric materials [7,19].

The following are some of the most recent studies in recent years on bio-based polymers using these three agri-food wastes [20,21,22,23,24,25,26]:

Klaai, L. et al. [20] studied the influence on the mechanical and thermal properties of fig seeds and olive husks as reinforcements for plastic by valorizing these agricultural wastes. The reinforcements were added to PLA in percentages ranging from 0 to 30%, with the result that olive husk reacted better thermally and mechanically than fig seeds, so it is interesting to continue studying the behavior of different residues of this type of fiber.

Chougan, M et al. [21] fabricated composites with wheat straw by functionalizing the surface of the straw with two nanomaterials, attapulgite nanoclay and graphene nanoplatelets, as they observed that if they increased the percentage (*v*/*v*) higher than 10%, there was a decrease in the tensile strength. 

Pereira, D.F. et al. [22] used rice husk reinforcement to make 3D printed filaments, treating the fibers with an alkaline NaOH treatment. They tested percentages from 0% to 20% *w*/*w*, the maximum amount the matrix could support, but only filaments containing no more than 10% *w*/*w* were FDM-processable. The alkaline treatment resulted in a reduction in *w*/*w* content. The studies by Pereira et al., which describe that chemical modification of lignin increases the production cost but has only a limited effect on the compatibility of lignin with the polymer, raise the question of whether it is worthwhile to modify the lignin structure or whether it is more interesting to control the particle size of the lignin; if this makes the process more expensive or reduces the percentage of lignin, which is ultimately the molecule that gives interesting properties to this type of material, it would not be worthwhile to carry it out. 

Authors such as Ariturk et al. [23], Lendvai [24] and Aliotta et al. [25] use lignocellulosic agro-residues like rapessed straw, hazelnut shell, cellulose or vermiculite with polylatic acid (PLA) to obtain biocomposites or green composites. They study their mechanical properties or use them to obtain 3D composite filaments or films which are state-of-the-art materials and are also biodegradable. There are also articles such as the study by Mohite et al. [26], which is a review of recycling of major agriculture crop residues and its application in the polymer industry, including PLA; this review is in the context of the waste-to-energy nexus.

In conclusion, the use of these lignin-rich wastes can make a positive contribution to the research begun by other authors on the revalorization of agri-food waste, with the novelty of focusing on the lignin properties of these reinforcements to try to contribute to improving the management of these wastes without ending up burning them. It is important to highlight that the composites incorporating this type of waste can be used in scarves or quilts in agriculture itself, thus contributing to a circular economy. Based on the above, the objective was to compare the composites obtained with the maximum percentage of reinforcement allowed by the matrix, so that it could be processed by extrusion and injection molding technologies without adjacent problems, as well as with virgin material, in order to observe how it affects the mechanical, thermal and morphological strength. The main objective is to study the impact of different lignin-rich waste composites, as well as commercial lignin itself, to know its “strengths” and “weaknesses” compared to virgin PLA, in order to propose solutions and applications where this type of waste can be used, contributing to the circular economy and sustainability.

## 2. Materials and Methods

### 2.1. Materials

Poly(lactic acid) (PLA), NaturePlast NP SF 141 extrusion grade, was purchased from NaturePlast from France. Lignin alkali was purchased from Sigma-Aldrich from Spain. Before use, the PLA and lignin (L) were first dried at 80 °C (4 h). 

On the other hand, olive pits, rice husk and wheat straw were obtained as waste from agri-food industries and provided by the partners of the AGROMATTER project, financed by the CDTI through the Ministry of Science and Innovation as part of the “Cervera” support for Centres of Technological Excellence CER-20211013. 

### 2.2. Development of PLA-Based Polymeric Materials with Agri-Food Waste through Melt Compounding

PLA NP SF 141 (extrusion grade) and its composites with agri-food waste additives were prepared by melt extrusion with the Rheoscam (Scamex), a single-screw extruder. The composites developed using this method were PLA/20% olive pits, PLA/20% husks and PLA/20% wheat straw. All the agri-food wastes described were ground with the same equipment, a RETSCH Ultra Centrifugal Mill, ZM200, 230 V/50 Hz, and sieved with a 0.5 mm heat-sensitive sieve. After sieving, they were dehumidified at 80 °C (4 h) and added to the PLA matrix as reinforcement. Table 1 shows the parameters used to obtain these composites.

### 2.3. Development of PLA-Based Polymeric Materials with Commercial Lignin through Melt Compounding 

This subsection includes the lignin/PLA composites developed and prepared by melt extrusion using Rheoscam (Scamex), a single-screw extruder. 

When developing a composite formulation, it is important to consider the maximum that the polymer matrix can support, depending on the additive in question. In other words, depending on the size, volume, density and compatibility of the additive with the matrix, the maximum percentage of the additive will be limited or not, depending on the nature of the additive. A clear example of this problem can be seen in the composites we have developed, where it can be seen that while agri-food waste can be added at 20% without any problem, commercial lignin can only be added at 6%. This is due to the fact that the integration of commercial lignin into the matrix is no longer homogeneous at this percentage. When this percentage is higher than 6%, the lignin cannot be integrated into the matrix as there is more dust than pellets and good phase integration is not achieved and the resulting polymer cannot be processed. Therefore, it has not been possible to add 20% lignin to PLA. However, there are numerous experiments where a certain percentage of additive can be added and the matrix will accept it well macroscopically, but the load on the matrix is still too high, resulting in a drop in mechanical properties due to stresses than can be observed in its morphology by SEM.

The following Table 2 summarizes the parameters used during the PLA/lignin composites’ extrusion. 

### 2.4. Agri-Food Waste and Lignin/PLA Composite Injection Molding

In order to be able to characterize the composites developed in the previous sections by extrusion, the injection of the necessary specimens was prepared for testing under ISO standards (ISO-527b and ISO-179). Since they are materials obtained on a laboratory scale, the injection was carried out with a Polytest (Ray-Ran) test sample preparation. The parameters used to obtain the specimens are shown in Table 3.

### 2.5. Characterization Methods

As all characterization tests were carried out in accordance with the applicable regulations, a minimum of 10 specimens were used, except for the study of thermal and morphological properties, where the test was regulated by grams (in case of DSC) or by different sections (morphological characterization).

#### 2.5.1. Determination of Density

The study to determine the density of the obtained composites was carried out according to the UNE-EN-ISO 1183-1 standard [27], specifically method A, that is, by immersing the injected sample in distilled water at a temperature of 23 °C. The equipment used for this test was an Alfa Mirge electronic densimeter supplied by Metrotec, S.A.

#### 2.5.2. Determination of Shore Hardness

Shore hardness was determined in accordance with the UNE-EN-ISO 868 standard [28], at a temperature of 23 °C and a relative humidity of 50 HR, on specimens measuring 80 mm × 10 mm × 4 mm and a Shore durometer IRHD Compact II (Digitest).

#### 2.5.3. Determination of Charpy Impact Strength

The Charpy impact test was performed according to UNE-EN-ISO 179-1 [29], at an impact speed of 2.9 m/s and nominal pendulum energy of 2 J, on standardized specimens injected with pellets, conditioned at 23 ± 2 °C and with a relative humidity of at least 50 ± 5% HR (16 h). Complete fracture of the specimens was achieved. This determination was carried out on a Metrotec, S.A., Impats 15 model, from the commercial brand ATS.

#### 2.5.4. Infrared Spectroscopy (FTIR) of Developed Composites

PLA, PLA/6% lignin, PLA/20% olive pit, PLA/20% rice husk and PLA/20% wheat straw were scanned 16 times by an FT-IR Bruker TENSOR 27 to estimate chemical bond differences in the range of 4.000–400 cm^−1^.

#### 2.5.5. Determination of Tensile Properties

To develop this test, standardized specimens were injected, with the dimensions described in UNE-EN-ISO 527b [30], using a test speed of 0.5 mm/s to obtain the elastic modulus and a speed of 25 mm/s to obtain the graphics related to the rip. To obtain the module, an extensometer was used and everything was carried out on the H10KS Tinius Olsen Universal Testing Machine, from Metrotec, S.A.

#### 2.5.6. Determination of Melt Flow Index (MFI) Properties

The determination of melt flow index (MFI) was also carried out following the instructions of the UNE-EN-ISO 1133-2 standard [31], conditioning the material with a dehumidification of 60 °C (3 h), with a preload without nominal load for 5 min and according to Method A, specified therein, at a temperature of 180 °C and a preheating time of 300 s. The nominal load use was 2.16 kg and the time between cuts was 3 s. The melt flow index study was performed on a Metrotec, S.A., MP600 Tinius Olsen plastometer, from the commercial brand ATS (Horsham, Pensilvania).

#### 2.5.7. Determination of Vicat Softening Temperature

Method B of the UNE-EN-ISO 306 standard [32] was used to determine the VICAT softening temperature, using parameters of 50 N and 50 °C/h on samples injected from pellets. The conditioning conditions were a temperature of 23 ± 2 °C and a relative humidity of 50 ± 10 HR (88 h) minimum. Heating was achieved by means of a thermal silicone oil bath. To obtain the VICAT softening temperature results, an Astfaar MP-3 from Metrotec, S.A., was used.

#### 2.5.8. Thermal Analysis

Thermal analysis was carried out by differential scanning calorimetry, DSC, using 1/200 System Mettler Toledo equipment. The composites obtained were previously dehumidified under the same conditions as the polymeric matrix. The test was carried out in accordance with UNE-EN-ISO 11357 standard [33], using 40 µL aluminum crucibles and a nitrogen flow rate of 50 mL/min. The following method was used because the polymer matrix is PLA: Step 1: 0 °C (5 min); Step 2: 0 °C–200 °C (10 °C/min); Step 3: 200 °C (10 °C/min); Step 4: 0 °C (5 min); and Step 5: 0 °C–200 °C (10 °C/min). The first heating was carried out in order to erase the thermal history of the material.

#### 2.5.9. Morphological Analysis

The morphology of the composites obtained was studied in the shear zone created by the blades of the pelletizer during extrusion, in order to observe the distribution of the filler in the polymeric matrix. This was carried out by scanning electron microscopy (SEM) using Carl Zeiss MERLIN equipment. Due to the polymeric nature of the material being studied, i.e., a non-conductive sample, it was considered that the sample should be coated with a conductive layer, in this case, gold, to minimize the risk of charging and thermal damage to the sample.

The following images show the developed composites in SEM at 30×, 500×, 1000× and 2.5k× magnifications to obtain an overview of the composites and the fillers and their homogeneity degree.

## 3. Results and Discussion

### 3.1. Physicochemical Characterization

#### 3.1.1. Determination of Density

The density values obtained remained practically constant after the addition of the byproducts, increasing only for wheat straw, but there was no significant variation. Table 4 summarizes all the values obtained. Composite materials with natural fibers usually have an average density between 1.3 and 1.5 g/cm^3^, this being one of their main advantages compared to other fibers, such as glass fiber, which has values of 2.6 g/cm^3^ [17].

#### 3.1.2. Determination of Shore Hardness

This property was affected by the addition of additives, especially in the case of olive pits, which showed a difference of 18.9%. In general, there was a decrease in Shore hardness for all composites developed compared to the virgin matrix, PLA. This decrease means a lower resistance to scratching and abrasion. However, the decrease in the value was not very significant, as all materials were in the “medium hardness range”, as can be seen in Table 5 below: 

#### 3.1.3. Determination of Charpy Impact Strength

In terms of Charpy impact strength, there was a clear tendency for this property to decrease with the addition of agri-food waste (Numerical values obtained for Charpy impact test on the composites obtained and on the virgin matrix are summarized in Table 6). However, this was not an abysmal difference, so the data obtained from the determination of the tensile properties of the material should be consulted before drawing any conclusions. Impact strength is related to toughness, which is the material’s resistance to fracture or the amount of energy required to propagate a crack. Authors such as Barreto et al. [19] have already described impact resistance values for PLA/rice husk composites with a percentage of 0–2% of additive, showing values in the range of 0.4–1.2 kJ/m. These values are not comparable with ours as the methodology is different; however, the trend in the materials is the same. Nyambo et al. [17], also performed impact tests for PLA/wheat straw materials, which document that the toughness decreases when fibers are added as additive to the PLA matrix. All this information is consistent because fibers, even when crushed and screened through 0.5 mm sieves, can agglomerate, causing stress concentrations that result in fracture of the material in the agglomeration zone, resulting in the observed property plunge for agri-food composites. 

#### 3.1.4. Infrared Spectroscopy of Developed Composites

**Figure 2:**
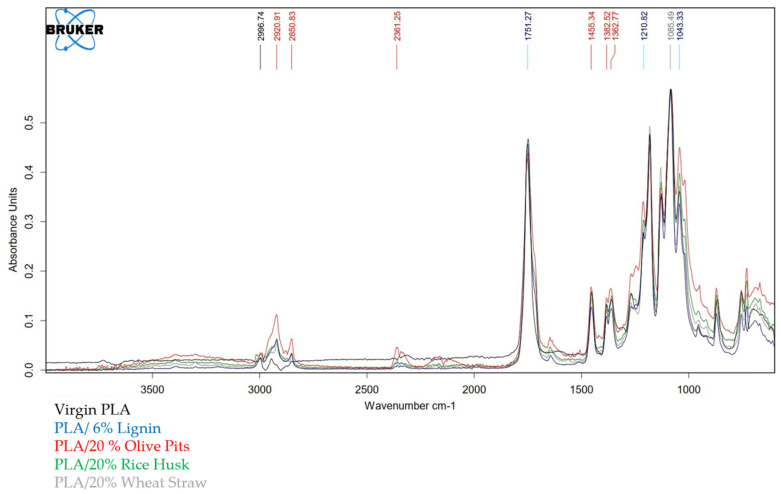
Agri-food waste/PLA composites, Lignin/PLA composites and virgin PLA infrared spectra.

Infrared spectroscopy was carried out to observe whether there were band shifts in pure PLA, PLA with lignin commercial additives and PLA with 20% agri-food waste (olive pits, rice husks and wheat straw). All materials showed some of the typical PLA bands described in the literature, such as the stretching of the –C-O bonds at 1085 cm^−1^, the bending of the CH_3_ groups at 1362 cm^−1^ and the symmetric and asymmetric vibrations of –CH at 2996 and 2920 cm^−1^, respectively. In all the additivated materials [34], a band appeared at 1646 cm^−1^ [35] that correlated to the carbonyl aldehyde group in lignin which did not appear in virgin PLA. Almost all samples showed a similar spectrum. In order to corroborate this information, composites spectra and polymer matrix especra are shown in Figure 2. 

#### 3.1.5. Determination of Tensile Properties

Table 7 summarizes the values obtained from the tensile test on the injected tensile specimens described in the previous sections. Firstly, the modulus of elasticity (Et) decreased more for the composite with agri-food waste. There was no trend, but it was decreases for the materials with olive pits and, to a lesser extent, for wheat straw, and it increased, exceeding the value of the virgin material, for rice husks. Impact strength decreased with the addition of any reinforcement, especially in the case of agri-food waste. This decrease in strength has already been reported in the literature. Lendvai, L. et al. [7] demonstrated a lower tensile strength of materials reinforced with rice husks. This lower strength could be attributed to inadequate interfacial bonding between the matrix and the additives. The “poor” bonding is not surprising given the rigid nature of the rice husks and their hydrophilicity compared to the hydrophobicity of the polymer.

#### 3.1.6. Determination of Melt Flow Index (MFI) Properties

The determination of the melt flow index was carried out on the pellets obtained after extrusion both for the pellets and for the composites with commercial lignin and agri-food residues and always in the same conditions: 180 °C and 2.16 kg of nominal load. This property increased significantly with the addition of fillers, with the value obtained for the composites being more than double that obtained for the virgin material (results are avaible in Table 8). It is worth noting that both olive pits and rice husks have the highest MFI values. Usually, MFI decreases on adding fillers. However, in 2017, Caicedo, C. et al. [36] explained how in the case of polypropylene, a reprocessing of this material produced an increase of up to 155% in the fifth reprocessing and this is due to the degradation of the material during the process, resulting in a decrease in the molecular weight of the polymer which causes an increase in the fluidity. Authors such as Hejna, A., [37] et al. also refer to an increase in fluency as a result of the incorporation of agri-food byproducts related to the chemical composition of the fillers, specifically the protein and lipid content of the skin. In their work, they point out that these compounds can act as plasticizers, improving the fluency and reducing the viscosity of the melt. However, they specify that in other similar work carried out by the same authors with polycaprolactone (PCL), this increase or decrease in fluency is not only related to the filler but also to the percentage added to the polymer matrix, as the incorporation of biofillers can sometimes limit the mobility of the polymer chains, making them responsible for their lower fluency [38]. 

#### 3.1.7. Determination of VICAT Softening Temperature

VICAT softening temperature determination indicates when a polymer starts to soften. This test showed that the material with commercial lignin and rice husk started to soften earlier than the other composites. This temperature gives us an idea of the zone determination interconnected to loss of material properties, such as *T_g_* for amorphous materials and *T_m_* for semi-crystalline materials. Table 9 summarizes the softening temperatures obtained for composites and virgin PLA, as shown below:

### 3.2. Thermal Analysis

Table 10 shows the melting points, melting enthalpies, glass transition temperatures, etc., obtained. 

A comparison with commercial lignin was also considered in order to observe if there were significant differences with respect to the commercial material; since authors such as Makri et al. [13] consider lignin to be a rigid molecule that can affect the thermal properties of polymers, we wanted to see if there were any noticeable differences between commercial lignin and added residues, as these contain other molecules in their structure in addition to lignin.

The thermal analysis of the developed composites was carried out using differential scanning calorimetry (DSC). Table 10 summarizes the thermal characteristics obtained for the virgin material (PLA) and the polymeric matrices with commercial lignin and PLA added with agri-food waste, including the glass transition temperature (*T_g_*), cold temperature (*T_cc_*), melting temperature (*T_m_*), crystallinity (*X_c_*), crystallization temperature (Δ*H_cc_*) and melting enthalpy (Δ*H_mm_*).

All composites showed their *T_g_*, *T_cc_* and subsequent melting. The addition of lignin as well as the residues added to the polymer matrix slightly decreased *T_g_* and increased *T_cc_* of PLA. These data are in agreement with those described in the literature. This decrease in T*_g_* can be attributed to interactions between lignin and PLA, possibly due to a plasticizing effect of a low-molecular-weight fraction of lignin. *T_m_* hardly varied and only decreased in the case of the composite containing olive pits. For the commercial lignin composite, a double peak was obtained. This kind of double peak has already been studied by other authors who state that it is due to the recrystallization and fusion of refined crystals, occurring only at low lignin loadings, since increasing this percentage would restrict the mobility of the chain [39].

*T_g_* is a non-complex phenomenon that depends on molecular interactions, steric effects, chain flexibility, branching and cross-linking density. The introduction of filler particles into the polymer matrix can increase, decrease or have no effect on the polymer matrix. The behavior of cold crystallization depends on many factors, but in general, it is the result of the interaction between kinetics, which generally hinder the chain mobility required for crystallization [40].

### 3.3. Morphological Analysis

**Figure 3:**
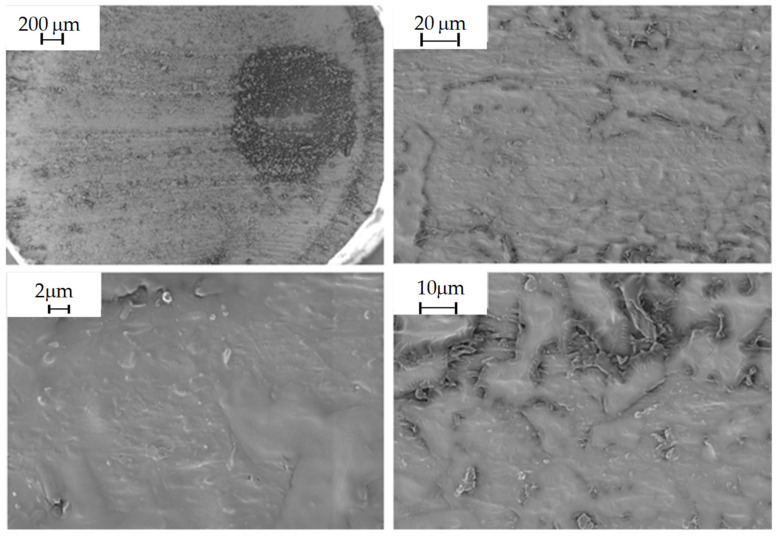
Virgin PLA observed at 30×, 500×, 1000× and 2.5k× magnifications by SEM.

**Figure 4:**
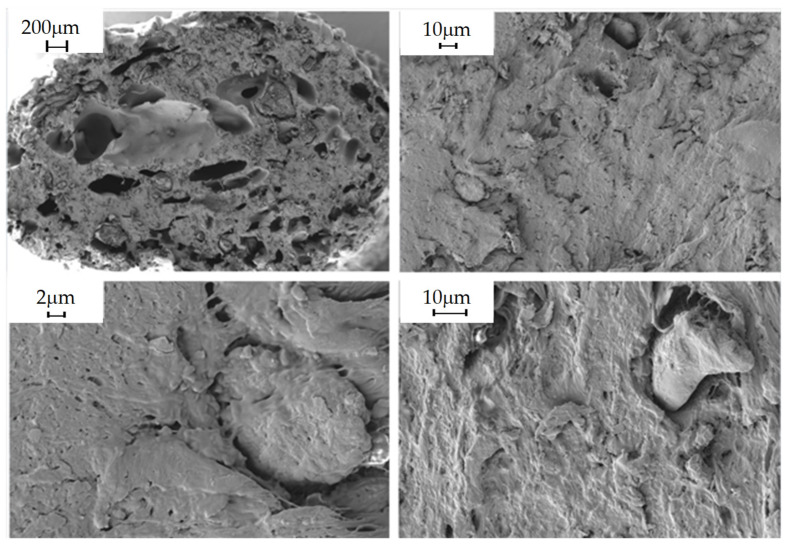
PLA/20% olive pit observed at 30×, 500×, 1000× and 2.5k× magnifications by SEM.

**Figure 5:**
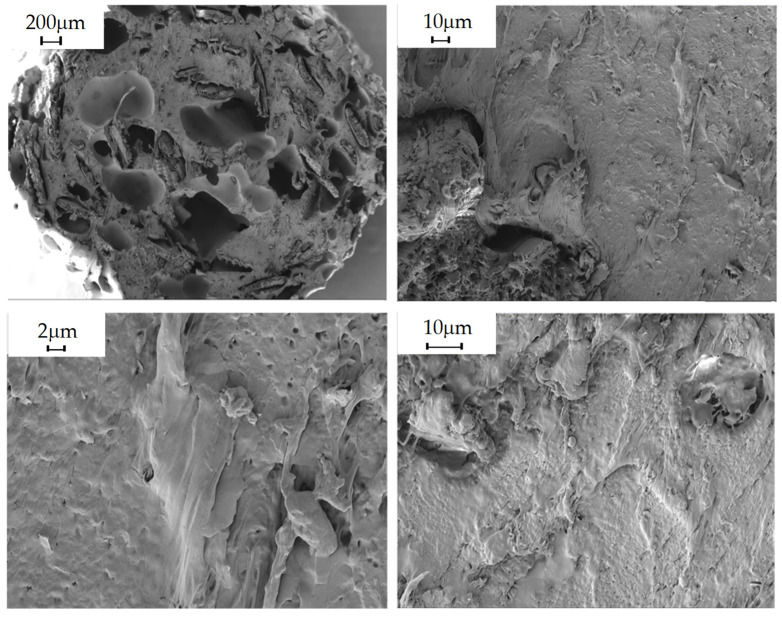
PLA/20% wheat straw observed at 30×, 500×, 1000× and 2.5k× magnifications by SEM.

**Figure 6:**
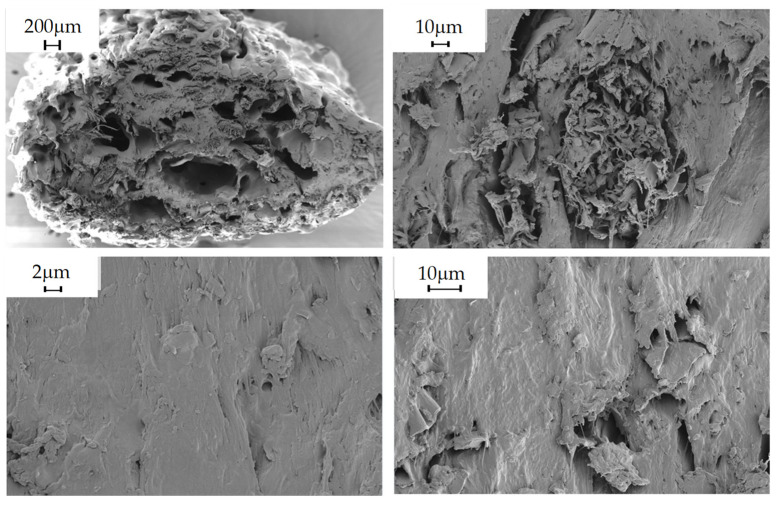
PLA/20% rice husk observed at 30×, 500×, 1000× and 2.5k× magnifications by SEM.

In this subsection, the figures obtained by SEM to evaluate the distribution and homogeneity of the agri-food wastes in the polymer matrix, are shown. Then, the virgin PLA, PLA/20% olive pit, PLA/20% wheat straw and PLA/20% rice husk, are shown in Figure 3, Figure 4, Figure 5 and Figure 6, respectively, at different magnifications indicated in each of them.

- Virgin PLA

- PLA/20% Olive Pit

- PLA/20% Wheat Straw

- PLA/20% Rice Husk

**Figure 7:**
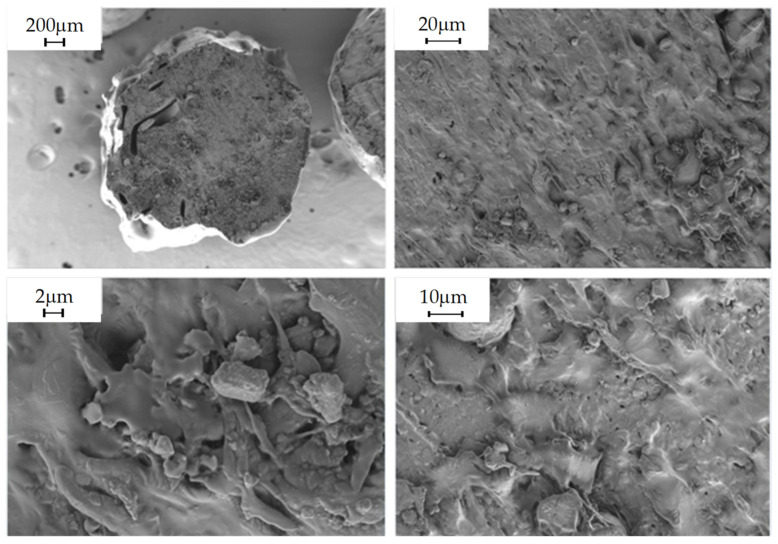
PLA/lignin (6%) observed at 30×, 500×, 1000× and 2.5k× magnifications by SEM.

It was also considered to observe the PLA/lignin composite with the maximum percentage of filler allowed in the matrix, in order to observe the differences in homogeneity that may occur. These differences can be seen in Figure 7.

Morphological properties are often overlooked because they are not given the same importance as mechanical properties. On the other hand, they can provide important information when evaluating a material. In these particular instances, the SEM images obtained for the composites, as well as those obtained for the virgin PLA, show that there is a great heterogeneity in the fillers, not only in their distribution, but also in their size variability, despite being crushed and sieved with the same mesh size. Consequently, despite the fact that the material was dehumidified in order to carry out the relevant extrusion processes, as well as the injection of specimens, there are numerous pores and voids in the materials, and it can even be observed that the additives are aggregated and heterogeneously distributed in the material, causing a clear fracture point that can cause a drop in the mechanical properties, both in terms of tensile strength and impact strength, since the presence of aggregates will cause the material to break [41].

## 4. Conclusions

The main conclusion is that lignin-rich agro-food wastes should be further investigated for use as reinforcement in biocomposites due to their very high potential. Despite being incorporated at relatively high percentages, these fillers have minimal effect on properties such as modulus and impact strength. In some cases, such as with rice husk, they even improve the modulus compared to virgin PLA. The reduction in Charpy impact strength observed in the more extreme cases can be mitigated by incorporating impact modifiers during polymer formulation and extrusion, or by chemical modification and/or functionalization of the fillers. However, the importance of mechanical properties is highly dependent on the intended application of the developed material. The increase in fluency when filler is added to the polymeric material is very interesting, because it is normally reduced. This is due to the fact that the PLA has been degraded during extrusion to obtain the composite, causing a shortening of the polymer chain, resulting in a decrease in the molecular weight of the polymer and therefore an increase in the fluency of the composite. In addition, as a consequence of agri-food waste chemical composition, they can act as a plasticizer. The literature cited in the Introduction and Results and Discussion sections of this article highlights the use of such polymers in food packaging to improve barrier properties, cosmetics and personal care, pharmacology and medicine and textiles and clothing. These studies have considered factors such as reduced tensile strength and impact resistance, indicating that such polymers are increasingly being used in applications such as cosmetic packaging.

## Figures and Tables

**Table 1:** Composites developed by melt extrusion with agri-food waste.

Material	Temperature Profile (°C)	Spindle Speed (rpm)	Pelletizer Speed (rpm)
PLA	121 130 141 145 145 145 145	160	28
PLA/20% olive pit	121 130 141 146 146 146 146	165	28
PLA/20% rice husk	121 130 141 146 146 146 146	165	28
PLA/20% wheat straw	125 135 144 149 149 150 154	160	28

**Table 2:** PLA/lignin composites developed by melt extrusion.

Material	Temperature Profile (°C)	Spindle Speed (rpm)	Pelletizer Speed (rpm)
PLA/6% lignin	175	15	7
	170		
	190		
	200		
	190		

**Table 3:** Injection molding parameters for developed composites.

Material	Injection Temperature (°C)	Mold Temperature (°C)	Heating Time (min)	Injection Pressure
PLA	170	65	5	7
PLA/20% olive pit	170	65	5	7
PLA/20% rice husk	175	65	5	7
PLA/20% wheat straw	180	65	5	7

**Table 4:** Density values obtained for composites and virgin PLA.

Material	Density (g/cm^3^)
PLA	1.252 ± 0.003
PLA/6% Lignin	1.230 ± 0.006
PLA/20% Olive Pit	1.249 ± 0.007
PLA/20% Wheat Straw	1.276 ± 0.002
PLA/20% Rice Husk	1.252 ± 0.005

**Table 5:** Shore hardness D/25 values obtained for composites and virgin PLA.

Material	Shore Hardness D/25
PLA	83.1 ± 0.39
PLA/6% Lignin	65.7 ± 0.37
PLA/20% Olive Pit	67.9 ± 0.36
PLA/20% Wheat Straw	70.1 ± 0.51
PLA/20% Rice Husk	70.2 ± 0.41

**Table 6:** Charpy impact strength values obtained for composites and virgin PLA.

Material	Charpy Impact (kJ/m^2^)
PLA	20.50 ± 1.87
PLA/6% Lignin	27.50 ± 2.24
PLA/20% Olive Pit	14.70 ± 1.77
PLA/20% Wheat Straw	13.34 ± 1.16
PLA/20% Rice Husk	13.20 ± 2.26

**Table 7:** Tensile values obtained for composites and virgin PLA.

Material	Elastic Modulus (MPa)	Strain at Break (%)	Stress at Break (σ*_b_*) (MPa)	Tensile Strength (σ*_m_*) (MPa)
PLA	2300 ± 169	159 ± 2.00	31.25 ± 0.73	54.85 ± 0.23
PLA/6% Lignin	1800 ± 070	9.55 ± 0.12	17.37 ± 0.54	23.82 ± 0.45
PLA/20% Olive Pit	2100 ± 175	27.40 ± 0.33	6.42 ± 0.66	9.55 ± 0.86
PLA/20% Wheat Straw	2200 ± 119	6.59 ± 0.39	9.37 ± 1.19	13.55 ± 0.73
PLA/20% Rice Husk	2500 ± 196	4.93 ± 0.81	10.32 ± 0.39	15.78 ± 0.56

**Table 8:** Fluency MFI (g/10 min) values obtained for composites and virgin PLA.

Material	Fluency MFI (g/10 min)
PLA	10.26 ± 0.005
PLA/6% Lignin	32.63 ± 0.001
PLA/20% Olive Pit	32.95 ± 0.004
PLA/20% Wheat Straw	20.67 ± 0.003
PLA/20% Rice Husk	32.63 ± 0.002

**Table 9:** VICAT softening temperatures (°C) obtained for composites and virgin PLA.

Material	VICAT Softening Temperature (°C)
PLA	64.43 ± 1.4
PLA/6% Lignin	46.00 ± 1.2
PLA/20% Olive Pit	61.00 ± 1.5
PLA/20% Wheat Straw	61.00 ± 1.6
PLA/20% Rice Husk	45.00 ± 1.6

**Table 10:** Thermal properties of the developed composites and virgin PLA obtained by differential scanning calorimetry (DSC).

Material	*T_g_* (°C)	*T_cc_* (°C)	Δ*H_cc_* (J/g)	*T_m_* (°C)	Δ*H_m_* (J/g)	*X_c_* (%)
Virgin PLA	63.35	80.48	10.22	153.03	13.02	3
PLA/6% Lignin *	62.85	81.72	6.34	141.25 153.09	19.33	15
PLA/20% Olive Pit	58.62	122.28	0.82	152.86	9.16	11
PLA/20% Wheat Straw	62.33	134.03	0.084	153.64	7.75	10
PLA/20% Rice Husk	61.89	133.37	0.027	154.60	11.62	15

* maximum percentage supported by the matrix.

## Data Availability

Data are contained within the article.
